# Characterization of Sera with Discordant Results from Reverse Sequence Screening for Syphilis

**DOI:** 10.1155/2013/269347

**Published:** 2012-12-27

**Authors:** Kyunghoon Lee, Hyewon Park, Eun Youn Roh, Sue Shin, Kyoung Un Park, Myoung Hee Park, Eun Young Song

**Affiliations:** Department of Laboratory Medicine, College of Medicine, Seoul National University, Seoul 110-744, Republic of Korea

## Abstract

Reverse sequence screening for syphilis (RSSS) (screening with treponemal tests, followed by confirmation with nontreponemal tests) has been increasingly adopted. CDC recommends confirmation of discordant results (reactive EIA/CIA and nonreactive nontreponemal test) with *Treponema pallidum* particle agglutination assay (TP-PA). We characterized sera with discordant results from RSSS with Architect Syphilis TP CIA. Among 15,713 screening tests using Architect Syphilis TP at Seoul National University Gangnam Center between October 2010 and May 2011, 260 (1.7%) showed reactive results. Rapid plasma reagin (RPR) and TP-PA were performed on 153 available sera among them. On sera with discordant results between Architect Syphilis TP and TP-PA, INNO-LIA Syphilis Score and FTA-ABS were performed. Among 153 sera, RPR was nonreactive in 126 (82.4%). Among them, TP-PA was positive in 103 (81.7%), indeterminate (±) in 7 (5.6%), and negative in 16 (12.7%). Out of 16 CIA(+)/RPR(−)/TP-PA(−) sera, INNO-LIA Syphilis Score and/or FTA-ABS were negative on 14 sera. Out of 7 CIA(+)/RPR(−)/TP-PA(±) sera, INNO-LIA Syphilis Score and FTA-ABS were positive/reactive in 6 sera. RSSS with confirmation by TP-PA on sera with discordant results between Architect Syphilis TP and RPR effectively delineated those discordant results and could be successfully adopted for routine checkup for syphilis.

## 1. Introduction

Because *Treponema pallidum*, which causes syphilis, cannot be cultured *in vitro*, serologic tests are the most frequently used methods to diagnose syphilis on suspected persons. These serologic tests are traditionally classified as “nontreponemal” and “treponemal”. Nontreponemal tests such as rapid plasma reagin (RPR) and venereal disease research laboratory (VDRL) detect antibodies directed against lipoidal antigens. Treponemal tests such as fluorescent treponemal antibody absorbed (FTA-ABS) test, *Treponema pallidum* particle agglutination assay (TP-PA), enzyme immunoassay (EIA), and chemiluminescence immunoassay (CIA) detect antibodies against individual or a mixture of specific *T. pallidum* proteins [1].

Whereas the traditional syphilis screening algorithm involves confirmation of reactive nontreponemal tests by treponemal tests in USA [2], it was recommended that a treponemal test should be used as a screening test followed by another type of treponemal test to confirm a reactive screening test in Europe [3, 4]. Recently, the Centers for Disease Control and Prevention (CDC) acknowledges the use of the reverse sequence screening for syphilis (RSSS) (treponemal tests for screening with confirmation of reactive results by nontreponemal tests) in addition to the traditional screening algorithm for syphilis [5, 6]. Specimens with reactive EIA/CIA results should be reflexively tested with a nontreponemal test (e.g., RPR or VDRL). If test results are discordant (reactive EIA/CIA, nonreactive RPR/VDRL), the specimen should be tested reflexively using the TP-PA test as a confirmatory treponemal test [6]. 

Although the TP-PA test was recommended as a most suitable second treponemal test for confirmation [6] considering its high sensitivity and specificity [7], the resolution and interpretation of those discordant sera (reactive EIA/CIA, nonreactive RPR/VDRL) are still challenging [8]. Some data showed there may not be a statistically significant difference in the performance of TP-PA and other treponemal tests [9]. Fourteen out of 26 discordant sera in syphilis screening in UK (reactive screening EIA and negative TP-PA) showed either reactive FTA-ABS or INNO-LIA syphilis score [10]. The aim of our study was to evaluate the overall efficacy of RSSS with Architect syphilis TP (Abbott, Wiesbaden, Germany) as a screening test and also to evaluate the accuracy of TP-PA test as a confirmation test on the discordant sera of RSSS.

## 2. Materials and Methods

### 2.1. Tests for Syphilis Screening and Confirmation

A total of 15,713 syphilis screening tests were performed using Architect Syphilis TP (Abbott, Wiesbaden, Germany)—an automated chemiluminescence immunoassay (CIA) at the Seoul National University Hospital Healthcare System Gangnam Center between 1 October 2010 and 31 May 2011. Two-hundred-sixty sera (1.7%) showed reactive results. Among them, 153 sera with sufficient amount of residual sera for additional tests were included in our study. Those sera were tested by RPR (Beckton Dickinson, Sparks, MD, USA) and with TP-PA (Fujirebio Inc., Tokyo, Japan) as a confirmatory second treponemal test. For 26 sera with discordant results among Architect Syphilis TP and TP-PA, the INNO-LIA syphilis score (Innogenetics, Gent, Belgium) and FTA-ABS (Scimedx, Denville, NJ, USA) test were performed to evaluate the utility of TP-PA. 

All the tests were performed and interpreted in accordance with the manufacturers' instructions delineated in the kit inserts. The Architect Syphilis TP is a chemiluminescent microparticle immunoassay for the quantitative detection of *T. pallidum*-specific antibodies using recombinant TP antigens such as TpN15, TpN17, and TpN47. RPR card antigen suspension is a carbon particle cardiolipin antigen which detects “reagin”, an antibody-like substance present in serum or plasma. The TP-PA uses gelatin particle carriers sensitized with purified *T. pallidum* (Nichols strain). Definite large ring with a rough multiform outer margin and peripheral agglutination was interpreted as “positive”, particles concentrated in the shape of a button in the center of the well as “negative”, and particles concentrated in the shape of a compact ring with a smooth round outer margin as “indeterminate” according to the instruction of manufacturer. 

The FTA-ABS detects circulating antibodies against the etiologic agent of syphilis, *T. pallidum*. The primary reaction involves antibodies which attach to antigens along the surface and internal structure of the microorganism. The INNO-LIA syphilis score is based on the enzyme immunoassay principle in which TpN47, TpN17, and TpN15 recombinant proteins and TmpA synthetic peptide are coated as discrete lines onto a nylon strip with plastic backing. Sera with more than 2 positive bands were interpreted as “positive” and sera with 1 positive band with a minimum intensity of 1 were interpreted as “indeterminate”. This study was approved by the institutional review board of Seoul National University Hospital (H-1104-127-360). 

### 2.2. Statistical Analysis

Mann-Whitney *U* test was used to compare the S/CO value of Architect Syphilis TP according to the results of RPR, TP-PA, or INNO-LIA syphilis score assays. ROC curve analysis of S/CO value of Architect Syphilis TP compared to TP-PA results was performed. All analyses used SPSS 18.0 statistical software (SPSS Inc., Chicago, IL, USA). *P* values <0.05 were considered significant.

## 3. Results

**Figure 1:**
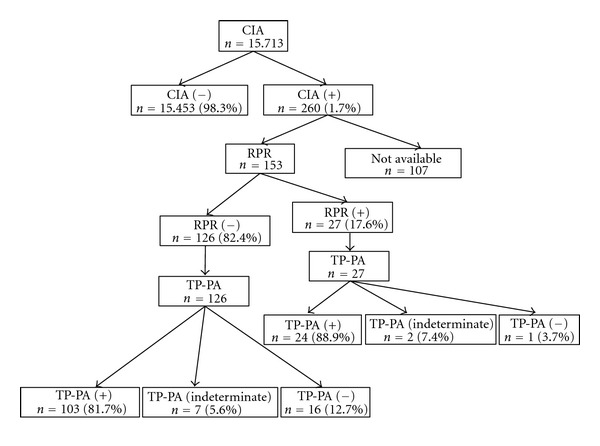
Results of reverse sequence screening algorithms for syphilis using chemiluminescence immunoassay (CIA) for initial screening, rapid plasma reagin (RPR) test, and *Treponema pallidum* particle agglutination assay (TP-PA) for confirmation.

Among the 153 available sera with CIA screening reactive results, RPR was reactive in 27 (17.6%) and nonreactive in 126 (82.4%) sera. Among the 126 CIA(+)/RPR(−) sera, TP-PA was positive in 103 (81.7%), indeterminate in 7 (5.6%), and negative in 16 (12.7%) sera. Among the 27 CIA(+)/RPR(+) sera, TP-PA was positive in 24 (88.9%), indeterminate in 2 (7.4%), and negative in 1 (3.7%) sera (Figure 1).

On 16 CIA(+)/RPR(−)/TP-PA(−) sera, the INNO-LIA syphilis score was positive in 1, indeterminate in 5, and negative in 10 (Table 1). For the 1 serum with INNO-LIA syphilis score positive results, FTA-ABS result was also reactive. And for the 10 sera with INNO-LIA syphilis score negative results, FTA-ABS results were all nonreactive. For the rest indeterminate 5 sera, FTA-ABS was reactive in 1 and nonreactive in 4. On 7 CIA(+)/RPR(−)/TP-PA(indeterminate) sera, both INNO-LIA syphilis score and FTA-ABS were positive/reactive in 6 sera and negative/nonreactive in 1 serum. On 1 CIA(+)/RPR(+)/TP-PA(−) serum, both INNO-LIA syphilis score and FTA-ABS were negative/nonreactive. On 2 CIA(+)/RPR(+)/TP-PA(indeterminate) sera, both INNO-LIA syphilis score and FTA-ABS were positive/reactive (Table 1).

**Figure 2:**
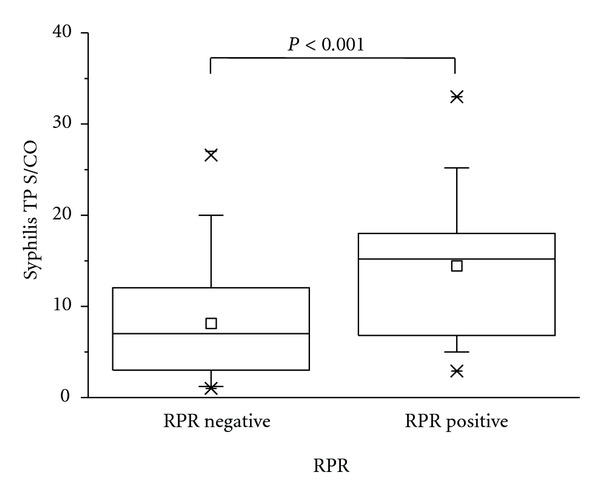
S/CO values of Architect Syphilis TP on 126 RPR(−) and 27 RPR(+) sera.

**Figure 3:**
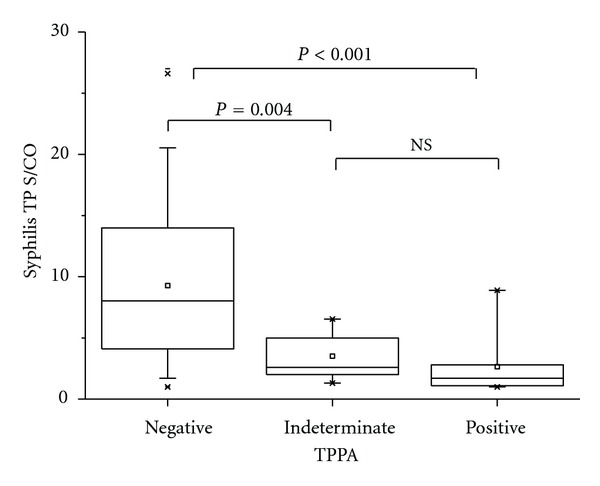
S/CO values of Architect Syphilis TP on 126 CIA(+)/RPR(−) sera according to TP-PA-positive (*N* = 103), indeterminate (*N* = 7), and negative (*N* = 16) results. NS: not significant.

**Figure 4:**
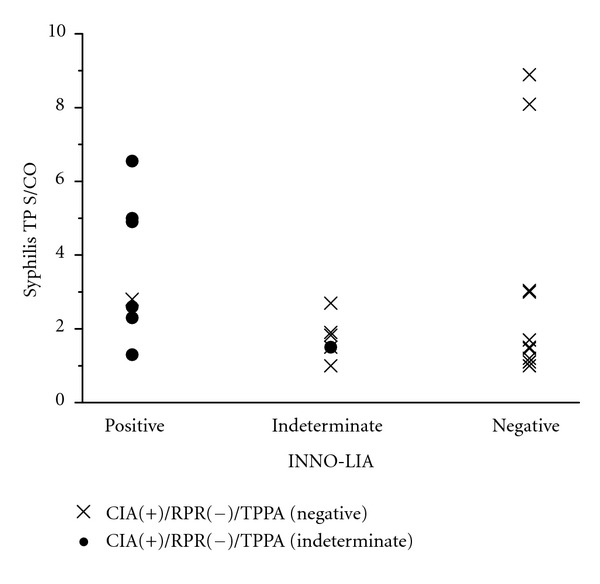
S/CO values of Architect Syphilis TP of 23 discordant sera between CIA and RPR/TP-PA according to the results of INNO-LIA syphilis score.

On 153 sera in our study, the signal to cut-off ratio (S/CO) value of Architect Syphilis TP in RPR reactive group (*N* = 27) was significantly higher than in RPR nonreactive group (*N* = 126) (*P* < 0.001) (Figure 2). On 126 discordant sera (Architect Syphilis TP reactive/RPR non-reactive), the S/CO value of TP-PA positive group (*N* = 103) was significantly higher than the S/CO value of TP-PA negative group (*n* = 16) (*P* < 0.001) and the TP-PA indeterminate group (*N* = 7) (*P* = 0.004) (Figure 3). On 23 discordant sera between Architect Syphilis TP and RPR/TP-PA (Syphilis TP reactive/RPR nonreactive/TP-PA indeterminate or negative), the S/CO values of Syphilis TP in INNO-LIA syphilis score-positive (*N* = 7), indeterminate (*N* = 6), and negative (*N* = 10) was not statistically different (Figure 4). 

**Figure 5:**
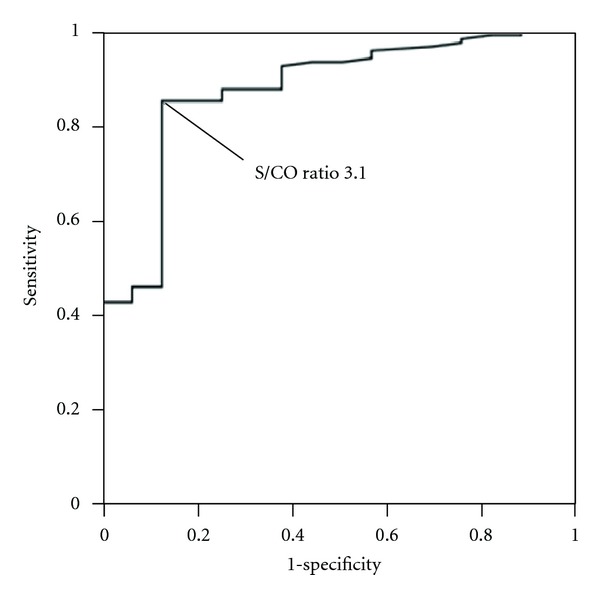
ROC curve of Architect Syphilis TP for prediction of TP-PA results on 126 CIA(+)/RPR(−) sera. The diagnostic sensitivity and specificity are 82.7% and 87.5% for an S/CO ratio of 3.1. The area under the curve (95% CI) is 0.872 (0.782–0.963).

For prediction of TP-PA results with S/CO value of Architect Syphilis TP, S/CO value of 3.1 showed good sensitivity (82.7%) and specificity (87.5%) with highest diagnostic efficacy (Figure 5). With the cut-off of S/CO 3.1, among 126 CIA(+)/RPR(−) sera, 32 sera would have been retested with TP-PA, and 2 sera would have been falsely reported as reactive. With the cut-off of S/CO 9.0, 82 sera would have been retested with TP-PA, and no sera would have been falsely reported as reactive. The area under the curve (95% CI) of S/CO value of Architect Syphilis TP was 0.872 (0.782–0.963).

## 4. Discussion

The efficiency of reverse sequence screening for syphilis (RSSS) could be dependent on the performance of screening treponemal test. Architect syphilis TP assay has been known to have an excellent sensitivity and specificity [11, 12]. In our study, from the RSSS using the Architect Syphilis TP CIA as a screening assay, the positive rate of CIA was 1.7% in our low-prevalence population, and the proportion of TP-PA-negative sera among 153 CIA-reactive sera was 10.5% (16/153). In USA, using two EIA assays (Trep-Chek, Trep-Sure EIA kit) and Liaison CIA assay as screening assays, the positive rate of CIA was 2.3% in low-prevalence population, and the proportion of TP-PA-negative sera among 2984 EIA/CIA-reactive sera was 24.7% (737/2984) [6]. Another study in Israel conducted on 12,235 low-prevalence population (4.0% positive rate of screening test) using Architect Syphilis TP as a screening test showed TP-PA-negative rate of 28.3% among 491 Architect Syphilis TP-reactive sera (139/491) [13]. TP-PA-negative rate was slightly lower in our study than in previous reports. It could be due to the difference of study population, prevalence of disease, or performance of screening assays detecting previously treated infection, but should be analyzed in further studies.

For the characterization of discordant sera between CIA and RPR, on 16 CIA(+)/RPR(−)/TP-PA(−) sera, 14 sera was INNO-LIA syphilis score and/or FTA-ABS nonreactive. On 7 CIA(+)/RPR(−)/TP-PA indeterminate sera, 6 sera was INNO-LIA syphilis score and FTA-ABS positive/reactive. Generally, TP-PA showed good performance to delineate discordant sera between CIA and RPR, although, TP-PA-indeterminate results should be confirmed with other treponemal tests. It supports the role of TP-PA in RSSS algorithms suggested by CDC considering the high performance of TP-PA. 

The increase of costs by RSSS has been reported recently [14]. To decrease the cost of confirmation test using TP-PA, we analyzed the S/CO value of Architect Syphilis TP correlates with the result of TP-PA. For HCV EIA/CIA, high S/CO value has been reported to be strongly associated with high positive predictive value [15, 16]. CDC recommended with high S/CO value, which shows 95% positive predictive value, there is no need for further confirmation test using RIBA or HCV-RNA test [17]. In our study, from the ROC analysis compared to TP-PA results, cut-off using S/CO of 3.1 on Architect Syphilis TP assay showed highest efficacy (sensitivity 82.7%, specificity 87.5%). With the cut-off of S/CO 3.1, among 126 CIA(+)/RPR(−) sera, 32 sera would have been retested with TP-PA, and 2 sera would have been falsely reported as reactive, which would have been most cost-effective. 

## 5. Conclusions

RSSS with confirmation by TP-PA on sera with discordant results between Architect Syphilis TP and RPR effectively delineated those discordant results and could be successfully adopted for routine checkup for syphilis. S/CO value of 3.1 on Architect Syphilis TP cost-effectively discriminated those sera which need further confirmations with TP-PA assays.

## Figures and Tables

**Table 1:** INNO-LIA syphilis score and FTA-ABS test results of 23 discordant sera between CIA and TP-PA results.

TP-PA	INNO-LIA syphilis score results (*N*)	FTA-ABS results (*N*)
RPR(−)/TP-PA(−) (*N* = 16)	Positive (1)	Reactive (1)
	Indeterminate (5)	Reactive (1) Non-reactive (4)
	Negative (10)	Non-reactive (10)

RPR(−)/TP-PA(±) (*N* = 7)	Positive (6)	Reactive (6)
	Indeterminate (1)	Non-reactive (1)

RPR(+)/TP-PA(−) (*N* = 1)	Negative (1)	Non-reactive (1)

RPR(+)/TP-PA(±) (*N* = 2)	Positive (2)	Reactive (2)

CIA: chemiluminescence immunoassay, RPR: rapid plasma regain, TP-PA: *Treponema pallidum* particle agglutination assay, FTA-ABS: fluorescent treponemal antibody absorbed, *N*: number, ±: indeterminate.
